# Integrative Analysis of miRNA and mRNA Expression Profiles Associated With Human Atrial Aging

**DOI:** 10.3389/fphys.2019.01226

**Published:** 2019-09-19

**Authors:** Yan Yao, Chenxi Jiang, Fan Wang, Han Yan, Deyong Long, Jinghua Zhao, Jiangang Wang, Chunxiao Zhang, Yang Li, Xiaoli Tian, Qing K. Wang, Gang Wu, Zhihui Zhang

**Affiliations:** ^1^Department of Cardiology, Beijing Anzhen Hospital, Capital Medical University, Beijing, China; ^2^Center for Cardiovascular Genetics, Department of Molecular Cardiology, Lerner Research Institute, Cleveland Clinic, Case Western Reserve University, Cleveland, OH, United States; ^3^Department of Human Population Genetics and Beijing Key Laboratory of Cardiometabolic Molecular Medicine, Institute of Molecular Medicine, Peking University, Beijing, China; ^4^Department of Cardiac Surgery, Beijing Anzhen Hospital, Capital Medical University, Beijing, China; ^5^Department of Cardiology, Renmin Hospital of Wuhan University, Wuhan, China; ^6^Department of Cardiology, The Third Xiangya Hospital of Central South University, Changsha, China

**Keywords:** atrial aging, miRNA, mRNA, interaction, signaling pathway

## Abstract

**Background:**

Limited findings have been reported to systematically study miRNA and mRNA expression profiles in aged human atria. In this study, we aimed to identify miRNAs, genes, and miRNA-mRNA interaction networks for human atrial aging (AA).

**Methods:**

Right atrial appendages from twelve patients who received aortic valve replacement were subjected to miRNA-seq and RNA-seq. All the patients were in sinus rhythm (SR) and stratified by age into four groups. Differential expression analysis was carried out to identify miRNAs and genes for human AA. The miRNA-mRNA interactions for human AA were identified by Pearson correlation analysis and miRNA target prediction programs.

**Results:**

Seven miRNAs (4 upregulation and 3 downregulation) and 42 genes (23 upregulation and 19 downregulation) were differentially expressed in human right atrial tissues between older samples and younger samples. Bioinformatic analysis identified 114 pairs of putative miRNA-mRNA interactions on AA and four types of correlation. Pathway enrichment analysis identified over 40 significant pathways and the top three pathways included rhythmic process (*P* = 7.5 × 10^–5^, *Q* = 0.034), senescence and autophagy in cancer (*P* = 9.0 × 10^–5^, *Q* = 0.034), and positive regulation of cytokine biosynthetic process (*P* = 1.1 × 10^–4^, *Q* = 0.034).

**Conclusion:**

Our study revealed novel miRNA-mRNA interaction networks and signaling pathways for AA, providing novel insights into the development of human AA. Future studies are needed to investigate the potential significance of these miRNA-mRNA interactions in human AA or AA-related cardiovascular diseases.

## Introduction

The incidence of atrial fibrillation (AF) increases dramatically in the older population (Schnabel et al., 2015). Aging results in a series of structural and physiological changes of the human atria, which may act as substrates to trigger AF (Pan et al., 2008; North and Sinclair, 2012). Advanced age causes aging-related atrial cardiomyopathy and facilitates the development of AF in patients without cardiovascular diseases (Goette et al., 2017). Moreover, aging significantly contributes to the high risk of ischemic stroke and heart failure in AF patients (Kirchhof et al., 2016). Therefore, aging has a close relationship with AF.

A growing number of studies have documented the evidence for the effects of aging on atrial structural remodeling, atrial electrophysiological remodeling, oxidative stress, and inflammation within the atria (Lin et al., 2018). However, few studies were reported to systematically study miRNA and mRNA expression profiles in aged human atria, which should provide new insights into the miRNA-mRNA regulatory networks during the progression of atrial aging (AA) (Bashan et al., 2012; Bartsch et al., 2015).

MicroRNA (miRNA) belongs to a class of small non-coding RNAs with 20–22 nucleotides in length. These small molecules exist in virtually all organisms and are evolutionarily conserved. By binding to their target mRNAs, miRNAs induce translational repression, mRNA deadenylation and mRNA decay at the post-transcriptional level (Liu et al., 2015). Many important biological processes are regulated by miRNAs, including proliferation, apoptosis, necrosis, autophagy, and stress responses (Ivanov et al., 2016). Some studies have revealed important contributions of miRNAs in cardiac tissues to the pathogenesis of AF, and the circulating miRNAs expressed in blood samples of AF patients may have potential value as diagnostic and prognostic markers (Luo et al., 2015; Zhou et al., 2018). However, the miRNA expression profile in AA is still unknown.

In this study, we enrolled sinus rhythm (SR) patients matched by age, gender and baseline cardiovascular diseases, and then performed high-throughput RNA sequencing (miRNA-seq and RNA-seq) to identify miRNA-mRNA interaction networks and AA-associated biological pathways. We found seven miRNAs and 42 genes that were differentially expressed among four age groups of patients. Interestingly, integrative analysis of miRNA-seq and mRNA-seq data identified 114 miRNA-mRNA interactions for AA and pathway analysis highlighted the rhythmic process and regulation of heart contraction in AA. Together, our study revealed novel miRNA-mRNA interaction networks and signaling pathways for AA (Ivanov et al., 2017).

## Materials and Methods

### Study Subjects

Patients who received open-heart surgery for aortic valve replacement in Beijing Anzhen Hospital between January 2017 and June 2017 were recruited for this study. All patients were in SR at the time of enrollment. Pre-operative two-dimensional color transthoracic echocardiography was performed routinely on the patients. Patients were excluded if diagnosed with or had a previous history of AF, hyper- or hypo- thyroidism, congenital heart diseases, rheumatic heart diseases, mitral valvular diseases or mitral prosthesis, left atrial diameter (LAD) >50 mm, uncontrolled hypertension (>160/90 mmHg), left ventricular dysfunction with an ejection fraction <40%, previous cardiac operations, malignancy, severe liver/renal dysfunction, and acute inflammatory diseases. Twelve patients (four females and eight males) were randomly selected for this study. The demographic and clinical features of the patients are shown in Supplementary Table S1.

The tip of right atrial appendage that was considered to be surgical waste was taken during the surgery. The human atrial tissues were immediately frozen in liquid nitrogen and saved for further experiments. This study was reviewed and approved by the Ethics Committee of Beijing Anzhen Hospital on human subject research and the use of human tissues. This study also complied with the guidelines set forth by the Declaration of Helsinki. Written informed consent was obtained from all patients.

### RNA Extraction

The total RNA was extracted from the frozen human right atrial appendages using TRIZOL reagent following the manufacturer’s protocol. The RNA yield and purity were determined using Agilent 2100 Bioanalyzer (Agilent Technologies, Santa Clara, CA, United States) and NanoDrop 2000 Spectrophotometer (Thermo Fisher Scientific, Waltham, MA, United States). All RNA samples passed quality control with an RNA integrity number of >7.

### miRNA-Seq

Genome-wide miRNA expression profiles of twelve human right atrial tissues were generated by miRNA-seq, which was carried out at the Biodynamic Optical Imaging Center (BIOPIC) in Peking University (Beijing, China). miRNA-seq followed the BIOPIC recommended protocol that was based on the NEBNext Multiplex Small RNA Library Prep Kit (Lot #E7560S). In brief, miRNAs were enriched from ∼2.5 μg of total RNA by gel selection (18–30 nt fragment size), and then ligated to 5′ and 3′ adapters and labeled with 6-nt barcodes. Barcoded miRNAs were subsequently reverse-transcribed and PCR-amplified. The resulted miRNA libraries were sequenced with Illumina HiSeq2500 platform. Around 10 million reads from 4 runs were generated for each sample.

Unless specified, default parameters were used in the following post-sequencing analysis. The overall quality of raw reads was evaluated using program FastQC (Andrews, 2010), and then raw reads were trimmed to remove adapter sequences using Cutadapt script (Martin, 2011). The cleaned reads were mapped to human genomes GRCh38 by Burrows-Wheeler Aligner (BWA) with parameters (-t 15, -n 1, -o 0, -e 0, -l 8, -k 0) (Li and Durbin, 2009). The mapped reads were sorted and indexed using samtools with BAM format (Li et al., 2009). The BAM data from multiple runs for the same sample were merged before miRNA quantification. Finally, a Python-based program HTSeq was used to quantify the expression level of known mature miRNAs curated in the recent human miRBase database (Griffiths-Jones et al., 2008; Anders et al., 2015).

### mRNA-Seq

For transcriptomic profiling, twelve human right atrial tissues were subjected to mRNA-seq according to the BIOPIC recommended workflow for mRNA-seq. A proportion (∼2.5 μg) of twelve isolated total RNA samples was used to generate cDNA libraries using Illumina TruSeq RNA Sample Preparation v2 Kit (Lot # RS-122-2001, RS-122-2002). The cDNA libraries were then sequenced by Illumina HiSeq2500 platform with pair-end 2 × 150 bp. mRNA-seq generated above 40 million reads with Q30 > 90% per sample on average. Raw mRNA-seq reads were first inspected for overall quality by FastQC and then cleaned by Cutadapt script (Andrews, 2010; Martin, 2011). The cleaned reads were mapped to human genome hg19/GRCh37 using the Subread aligner (Liao et al., 2013), which allows fast and efficient mapping for short reads less than160 bp. Finally, gene-level quantification was performed using featureCounts (Liao et al., 2014), which is a reads summarization program to efficiently count the mapped reads for genomic features (i.e., genes and exons). In this study, all human genes under genome build hg19 were retrieved via R annotation package org.Hs.eg.db (see the link in the section “Data Availability Statement”).

### Differential Expression Analysis

Differential expression analysis was performed to identify miRNAs and genes that were either upregulated or downregulated in atrial tissues of older samples compared to that of younger samples. The miRNA-seq and mRNA-seq data were analyzed by R package edger (Robinson et al., 2010; McCarthy et al., 2012), which employs a negative binomial generalized linear model with likelihood ratio test (glmLRT) to compare read counts of each miRNA or gene between two conditions. Raw counts were adjusted by library size to account for sample-specific effects. The count-per-million at log2 scale, denoted as LogCPM, was computed for visualization of miRNA/gene abundance, heatmap clustering analysis, and miRNA-mRNA correlation analysis.

To reduce false positive signals and obtain more conserved results, twelve patients in SR were assigned into four groups: SR40 (38–42 years old), SR50 (48–52 years old), SR60 (58–62 years old) and SR70 (68–72 years old). Each age group included one female and two males. Then, glmLRT was run for SR60 vs. SR40 and SR70 vs. SR50. miRNAs and genes showing *P* < 0.05 and the same direction in the above two tests were reported in this study. After that, using *P* values from the above two tests, a meta-analysis with Fisher’s method was carried out to evaluate the overall significance of the identified miRNAs and genes (Chang et al., 2013). For the multiple testing issue, *P* values were adjusted by the false discovery rate (FDR) method (Dabney et al., 2010). For the top AA-miRNAs and AA-genes, their linear trends with aging were examined using simple linear regression. The regression coefficients, confidence intervals and *P* values were calculated using R v3.0.0^1^.

### miRNA-mRNA Interaction Analysis

Given the availability of both miRNA-seq and mRNA-seq data on 12 patients (Supplementary Table S1), Pearson correlation test was used to examine pair-wise correlations between seven AA-miRNAs and 23,346 genes. In this study, a valid miRNA-mRNA interaction was reported based on the following conditions: (i) both miRNA and gene were associated with AA, i.e., FDR < 0.05; (ii) miRNA significantly correlated with gene expression, i.e., *P* value < 0.05; (iii) directions of effect sizes of miRNA and gene on aging were consistent with that from pair-wise correlation analysis. For each AA-miRNA, the target genes were predicted using program multiMiR (Ru et al., 2014), which integrated 14 miRNA-mRNA interaction databases such as TargetScan (Lewis et al., 2005), miRDB (Wong and Wang, 2015), miRanda (Betel et al., 2010), miRTarBase (Chou et al., 2018), and others. If any valid miRNA-mRNA pair for AA was predicted by multiMiR, it was classified as a direct interaction; otherwise, it was an indirect interaction.

### miRNA-mRNA Network and Pathway Enrichment Analysis

One hundred fourteen miRNA-mRNA interactions, including eight direction interactions, were interconnected in a network using program Cytoscape^2^. All genes in the network were analyzed by program clusterProfiler (Yu et al., 2012) to determine whether AA-genes were enriched in biological pathways curated in biological knowledge databases such as Gene Ontology (GO), Kyoto Encyclopedia of Genes and Genomes (KEGG) and WikiPathways. *P* values from enrichment analysis were adjusted using the FDR method.

### Real-Time RT-PCR Analysis

Three miRNAs (miR-23a-5p, miR-1263, and miR-6514-3p) were selected to confirm the results of miRNA-seq by qRT-PCR analysis. miRNAs were converted to cDNA using Mir-X^TM^ miRNA First-Strand Synthesis Kit (Thermo Fisher Scientific, Waltham, United States) and subjected to SYBR^®^ qRT-PCR analysis (Takara, Osaka, Japan). The qRT-PCR was performed with Applied Biosystems 7500 (Thermo Fisher Scientific, Waltham, MA, United States). U6 was used as a reference gene for normalizing miRNA expression. Primer sequences were as follows: miR-23a-5p: 5′-GGG GTT CCT GGG GAT GG-3′, miR-1263: 5′-CAT GGTA CCC TGG CAT ACT GAG T-3′, miR-6514-3p: 5′-CAA ACA AAC ATG GTG CAC TTC TT-3′.

### Data Availability and Public Resources

The data supporting the findings of this study, including the full summary statistics of miRNA-seq and mRNA-seq analysis, is available within this manuscript as well as Supplementary Material. The source codes and analytic pipeline used for processing RNA-seq data are open to the public at https://github.com/zhenyisong/wanglab.code. We confirm that the raw sequencing data is immediately available to all researchers upon request with approval from the institutional research committee. Mouse miRNA microarray data for cardiac aging (GEO Accession: GSE43556) were analyzed using GEOquery (Davis and Meltzer, 2007) and Limma (Ritchie et al., 2015). MiRBase: ftp://mirbase.org/pub/mirbase; Human Gene Annotation: 10.18129/B9.bioc.org.Hs.eg.db; GO: http://www.geneontology.org/; KEGG: http://www.genome.jp/kegg/pathway.html; WikiPathways: https://www.Wikipathways.org/index.php/WikiPathways.

## Results

### miRNA-Seq Analysis Identified 7 miRNAs Associated With Human AA

**FIGURE 1:**
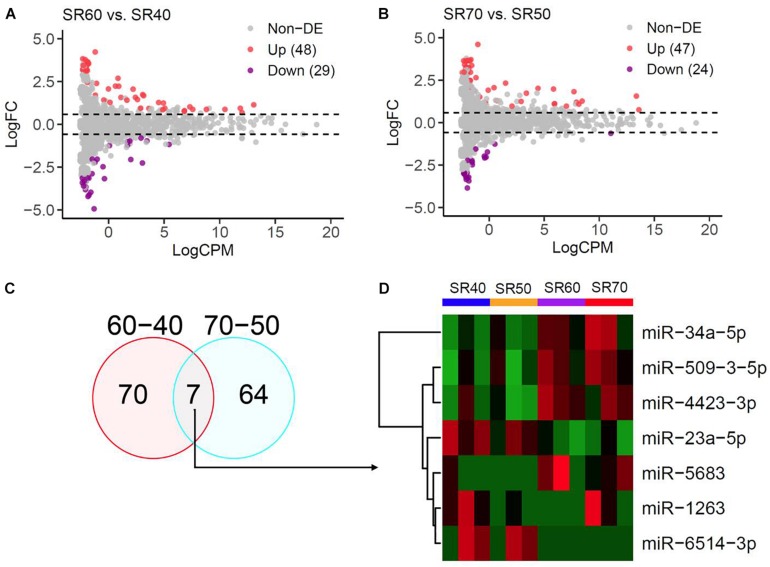
miRNA-seq analysis of human atrial samples with SR. **(A)** Differential expression analysis of 1,482 miRNAs between 60 and 40 years old group. *X*-axis, miRNA expression was quantified as count per million at log2 scale. *Y*-axis, fold change (SR60/SR40) at log2 scale. **(B)** Differential expression analysis of 1,482 miRNAs between 70 years old group and 50 years. **(C)** Venn diagram showing significant miRNAs from the above two comparisons at nominal *P* < 0.05. **(D)** Heatmap and hierarchical structure of seven overlapping miRNAs across 12 human atrial samples.

Twelve patients in SR from four age groups were analyzed in this study. The individual-level phenotypic features are shown in Supplementary Table S1. A total of 1,482 mature miRNAs were identified by miRNA-seq, and the overall quality and distribution across 12 SR samples are shown in Supplementary Figure S1. To reduce false positive signals, we carried out differential expression analysis in two sets. In the first set, compared with the SR40 group, we identified 48 upregulated and 29 downregulated miRNAs in SR60 group at *P* < 0.05 (Figure 1A). In the second set, compared to SR50, we found 47 upregulated and 24 downregulated miRNAs in SR70 group at *P* < 0.05 (Figure 1B). Summary statistics of all AA-miRNAs in each set are shown in Supplementary Tables S2, S3.

**FIGURE 2:**
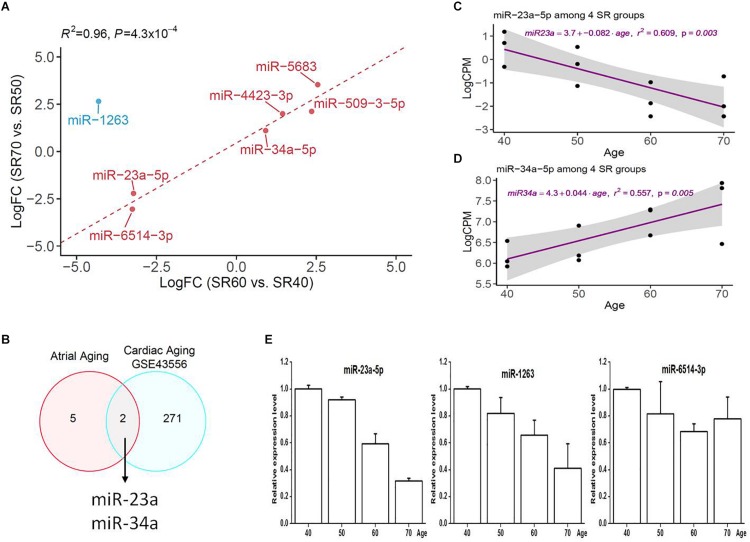
Comparative analysis of seven human AA-associated miRNAs. **(A)** Correlation analysis of effect size of miRNAs from SR60 vs. SR40, and SR70 vs. SR50. LogFC, fold change (SR60/SR40 or SR70/SR50) at log2 scale. **(B)** Venn diagram showing two miRNAs overlapping with cardiac aging-related miRNAs [GSE433556, false discovery rate (FDR) < 0.05]. **(C,D)** Linear regression analysis of miR-23a-5p and miR-34a-5p with age. The miRNA expression was quantified as count per million at log2 scale (*Y*-axis). For each miRNA, linear tread and 95% confidence interval were shown as purple line and a gray area. **(E)** Independent qRT-PCR replication of three miRNAs among four groups.

Seven miRNAs were identified by two subsets (Figure 1C), and heatmap plot showed a clear expression pattern that matched with the status of human age (Figure 1D). Moreover, we found that effect sizes of 7 miRNA were highly consistent between two sets (Figure 2A and Table 1) with the exception of miR-1263. Interestingly, we found that two miRNAs (miR-23a and miR-34a) have been linked to cardiac aging (GSE43556, Figure 2B). In the secondary analyses, we assessed the linear trends of AA-miRNAs in the combined set (from 40 to 70 years old). Not surprisingly, miR-23a-5p and miR34a-5p showed a strong linear correlation with age (Figures 2C,D and Supplementary Figure S2).

**TABLE 1:** miRNA-seq Identified 7 miRNAs Associated with Human AA.

**miRNA**	**SR60 vs. SR40**				**SR70 vs. SR50**				**Meta-analysis**	
			
	**LogFC**	**LogCPM**	**LR**	***P***	**LogFC**	**LogCPM**	**LR**	***P***	**Direction**	***P*_Fisher_**
hsa-miR-23a-5p	–3.22	–0.36	13.23	2.8E−04	–2.22	–0.36	6.49	0.011	−−	4.1E−05
hsa-miR-1263	4.31	1.70	6.97	0.008	2.65	1.70	4.34	0.037	− +	0.003
hsa-miR-509-3-5p	2.36	0.43	5.82	0.016	2.12	0.43	5.05	0.025	++	0.003
hsa-miR-34a-5p	0.92	6.92	4.41	0.036	1.10	6.92	6.34	0.012	++	0.004
hsa-miR-4423-3p	1.45	0.25	3.92	0.048	1.99	0.25	6.13	0.013	++	0.005
hsa-miR-5683	2.55	1.80	3.94	0.047	3.53	1.80	5.00	0.025	+ +	0.009
hsa-miR-6514-3p	3.26	2.12	4.10	0.043	3.05	2.12	4.02	0.045	−−	0.014

*LogFC, fold change at log2 scale. LogCPM, count per million at log2 scale. LR, likelihood ratio statistics. *P*, *P* values calculated by GLM-LRT model. *P*_*fisher*_, *P* value of Fisher test. Direction, miRNA expression was higher (+), or lower (−) in older group compared with younger group.*

Three miRNAs (miR-23a-5p, miR-1263, and miR-6514-3p) were selected for an independent replication by qRT-PCR; the results were shown in Figure 2E. Note that we did not exclude miR-1263 from downstream analysis as it was successfully confirmed by qRT-PCR analysis.

### mRNA-Seq Based Meta-Analysis Identified 42 Genes Associated With Human AA

**FIGURE 3:**
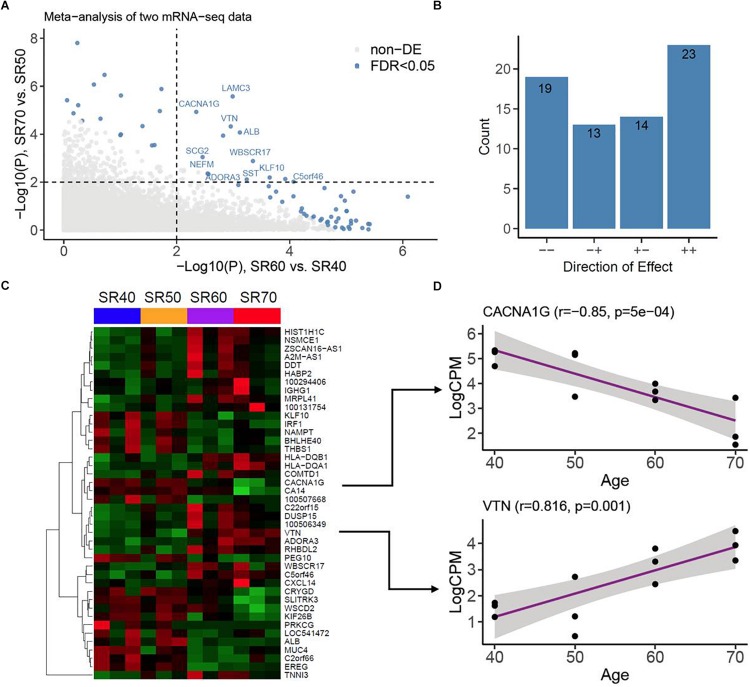
mRNA-seq analysis of human atrial samples. **(A)** Meta-analysis of two mRNA-seq datasets. *X*-axis, −log10 (*P* value) calculated from differential analysis between SR60 and SR40. *Y*-axis, −log10 (*P* value) calculated from differential analysis between SR70 and SR50. Dots with steel blue color indicate genes (or mRNAs) significantly associated with human AA at FDR < 0.05. **(B)** Counts of genes with opposite (−+ and +) and consistent effects (− and ++). **(C)** Heatmap and hierarchical structure of 42 AA-associated genes across 12 human atrial samples. Each row indicated a gene, its Entrez ID or official symbol was shown at right side. **(D)** Examples of top-ranked genes (*CACNA1G and VTN*) showing strong linear trends with human age. Pearson Correlation coefficient (*r*) and the corresponding *P* value were shown at the top.

With mRNA-seq, transcriptomic profiles of 23,346 genes were obtained from same 12 SR samples. The overall quality and distribution across 12 samples are shown in Supplementary Figure S3. We then identified genes associated with human AA with the similar strategy as for miRNA-seq. After analyzing two subsets of mRNA-seq, we performed a meta-analysis of 2 subsets of results with the Fisher’s test, which combined 2 sets of *P* values into one set (Figure 3A). We found 69 genes associated with AA at FDR < 0.05. Of these, 42 genes (23 upregulation and 19 downregulation) were consistent between two sets (Figure 3B and Supplementary Tables S4, S5). Heatmap plot showed a clear expression pattern that matched with the status of human age (Figure 3C). We also assessed the linear trends of 42 genes in the combined set. Two examples (*CACNA1G* and *VTN*) are shown in Figure 3D.

### Integrative Analysis Identified miRNA-mRNA Interaction Networks for AA

**FIGURE 4:**
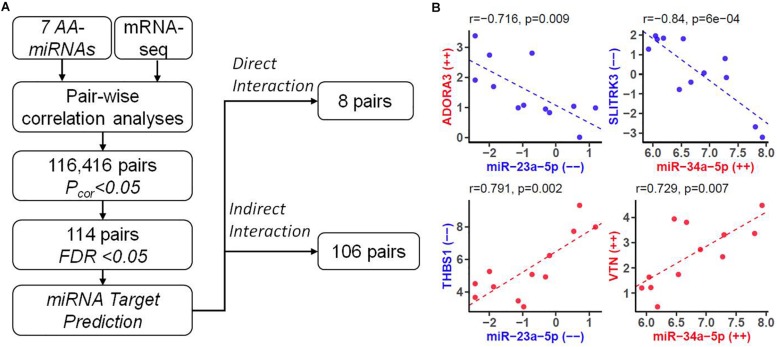
Identifying downstream genes of AA-associated miRNAs. **(A)** Flowchart of predicting miRNA-mRNA pairs for human AA by integrating mRNA-seq data with bioinformatics analysis. FDR, false discovery rate. **(B)** Four types of miRNA-mRNA interactions. In each plot, X-axis represented expression levels (count per million at log2 scale) of a miRNA, and Y-axis represented expression levels of a gene. Labels with “–” or “++” indicated decreased or increased expression in older group compared to younger group. Such effect size were also highlighted by color (color, down-regulation; red, up-regulation). Pearson Correlation coefficient (*r*) and the corresponding *P* value were shown at the top.

We established an effective pipeline to identify miRNA-mRNA interaction for AA. The flowchart of integrative analysis was displayed in Figure 4A. For each of 7 AA-miRNAs (Table 1), we analyzed its correlation with all 23,346 genes generated by mRNA-seq. When looking at distributions of genome-wide miRNA-mRNA interactions, we found that three miRNAs (miR34a-5p, miR-4423-3p, and miR-509-3-5p) showed negative correlations with their predicted targets (Supplementary Figure S4). A total of 116,416 pairs were identified by Pearson correlation tests with *P* < 0.05. Enrichment analysis also showed that the predicted targets of 4 miRNAs were highly overlapped with 42 AA-genes (Table 2). Therefore, 122 pairs were found by restricting to the most significant 42 AA-genes (FDR < 0.05). Eight pairs were excluded because gene names were not annotated from human gene annotation database.

**TABLE 2:** Prediction of AA-miRNA Downstream Targets.

**miRNA**	**Direct Targets**				**Indirect Targets**			
		
	**N1**	**AA-mRNA**	**FC**	***P*_hyper_**	**N2**	**AA-mRNA**	**FC**	***P*_hyper_**
hsa-miR-1263	13	0	0.00	1.00	921	0	0.00	1.00
hsa-miR-23a-5p	131	1	4.24	0.007	3,736	25	3.72	0.00
hsa-miR-34a-5p	618	5	4.50	0.00	2,207	19	4.79	0.00
hsa-miR-4423-3p	94	0	0.00	1.00	2,008	20	5.81	0.00
hsa-miR-509-3-5p	411	2	2.70	1.9 × 10^–5^	3,111	27	4.82	0.00
hsa-miR-5683	4	0	0.00	1.00	306	1	1.86	0.009
hsa-miR-6514-3p	45	0	0.00	1.00	2,146	15	3.89	0.00

*N1, number of direct targets of miRNAs; N2, number of indirect targets of miRNAs. AA-mRNA, number of targets who were associated with AA by mRNA-seq at FDR < 0.05. FC, enrichment fold. *P*_*hyper*_, *P* value from hypergenometric test.*

We analyzed the remaining 114 miRNA-mRNA pairs by taking the direction of effects of miRNAs and genes on human AA and obtained four types of correlation (Figure 4B). Eight pairs were found with direct interaction (Table 3), which was defined as the gene of a miRNA-mRNA pair predicted to be a target of miRNA and also negatively correlated with the expression of miRNA. The other 106 pairs demonstrated indirect interaction (Supplementary Table S6).

**TABLE 3:** List of AA-miRNA and Downstream Direct Targets.

**miRNA**	**Gene**	**miRNA-seq**		**miRNA-mRNA correlation**		**mRNA-seq**		
				
		**Direction**	***P*_Fisher_**	***r***	***P*_cor_**	**Direction**	***P*_Fisher_**	**FDR**
hsa-miR-23a-5p	*ADORA3*	–	4.1E−05	−0.72	8.8E−03	++	1.6E−04	0.050
hsa-miR-34a-5p	*SLITRK3*	++	3.7E−03	−0.84	6.4E−04	−−	4.5E−07	0.003
hsa-miR-34a-5p	*THBS1*	++	3.7E−03	−0.61	3.5E−02	−−	6.0E−07	0.003
hsa-miR-34a-5p	*EREG*	++	3.7E−03	−0.79	2.2E−03	−−	3.1E−06	0.007
hsa-miR-34a-5p	*IRF1*	++	3.7E−03	−0.65	2.3E−02	−−	4.7E−05	0.031
hsa-miR-34a-5p	*PEG10*	+ +	3.7E−03	−0.74	5.5E−03	−−	1.3E−04	0.048
hsa-miR-509-3-5p	*THBS1*	++	3.5E−03	−0.76	3.8E−03	−−	6.0E−07	0.003
hsa-miR-509-3-5p	*KLF10*	++	3.5E−03	−0.68	1.5E−02	−−	2.1E−05	0.022

**r*, correlation coefficient. *P*_*cor*_, *P* value of miRNA-mRNA correlation analysis. *P*_*fisher*_, *P* value of meta-analysis with Fisher test. Direction, miRNA expression was higher (+), or lower (−) in older group compared with younger group. FDR, false discovery rate.*

**FIGURE 5:**
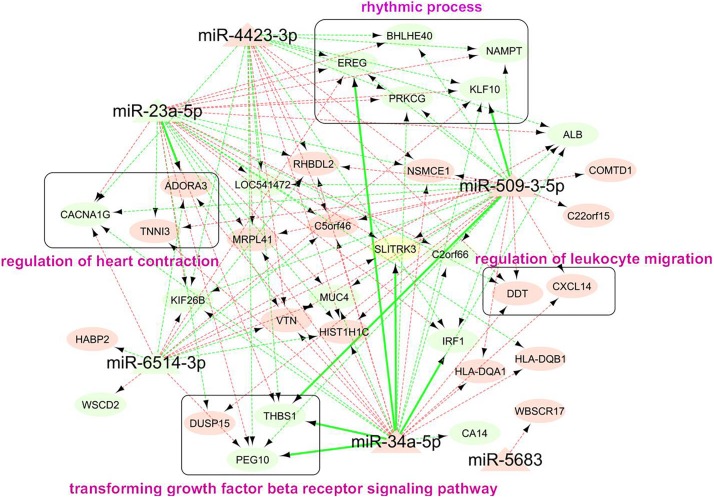
Network analysis of 114 miRNA-mRNA pairs associated with human AA. Each node represented a miRNA (triangle shape) or gene (oval shape). The color of node indicated whether the expression of a miRNA or gene increased (light red) or decreased (light green) in older group. The color of edge indicated the negative (light green) or positive (light green) correlation between miRNA and downstream gene. Edge with sold line indicated direction interaction between miRNA and gene, i.e., gene was a predicted target of miRNA and it negatively correlated with expression of miRNA. Edge with dashed line indicated miRNA-mRNA pairs with indirect correlation, that said, the gene strongly correlated with expression of miRNA (*P*cor < 0.05) and AA (FDR < 0.05). The genes in round rectangle were highlighted by gene set enrichment analysis (Supplementary Table S3).

Our miRNA-mRNA analysis identified seven direct target genes for six miRNAs, which accounted for 16.7% (7/42) of top-ranked AA-genes. The majority of 42 AA-genes were indirect targets of 6 miRNAs. A network of 6 AA-miRNA and 34 targets is shown in Figure 5. Pathway analysis identified over 40 significant pathways (Supplementary Table S7). Top pathways underlying AA included rhythmic process (*P* = 7.5 × 10^–5^, *Q* = 0.034), senescence and autophagy in cancer (*P* = 9.0 × 10^–5^, *Q* = 0.034), positive regulation of cytokine biosynthetic process (*P* = 1.1 × 10^–4^, *Q* = 0.034), circadian rhythm (*P* = 2.5 × 10^–4^, *Q* = 0.034), and transforming growth factor-beta(TGF-β) receptor signaling pathway (*P* = 2.5 × 10^–4^, *Q* = 0.034).

## Discussion

Herein, we reported the first study to systematically analyze miRNA and mRNA expression profiles of right atrial tissues across human samples with different age stages. With miRNA-seq and mRNA-seq, we found seven miRNAs and 42 genes whose expression levels significantly correlated with human AA. Integrative analysis identified 114 interactions between seven AA-miRNAs and 42 AA-genes. Pathway enrichment analysis of all genes regulated by AA-miRNAs highlighted rhythmic process and regulation of heart contraction in human AA.

One important finding of our study was the identification of seven miRNAs significantly associated with human AA by taking the advantages of miRNA-seq and reciprocal replication analysis of two sets of human right atrial samples (Figure 1). In each set, older samples (SR60 or SR70) and younger samples (SR40 or SR50) were matched by gender and clinical baseline features to minimize the influence of confounding factors on differential expression analysis (Supplementary Table S1). Four upregulated miRNAs (miR-34a-5p, miR-5683, miR-4423-3p, and miR-509-3-5p) and two downregulated miRNAs (miR-23a-5p and miR-6514-3p) were found to show a consistent pattern between two sets of samples (Table 1). The exception is miR-1263, whose expression pattern was initially opposite between two sets of miRNA-seq data but later confirmed to be downregulated by independent qRT-PCR analysis (Figure 2). Of seven AA-miRNAs, miR-34a was previously reported to be upregulated in aging mouse heart. Boon et al. (2013) showed that miR-34a contributed to cardiac aging by regulating telomere shortening, DNA damage response and cardiomyocyte apoptosis. miR-34a was also upregulated in various vascular aging models by decreasing sirtuin1 expression (Emanueli and Thum, 2013; Yu et al., 2015). Atorvastatin treatment could enhance sirtuin1 expression via inhibition of miR-34a, possibly contributing to the beneficial effects of atorvastatin on endothelial cell function (Tabuchi et al., 2012). Our study provided further evidence that miR-34a is a key regulator in both cardiac aging and vascular aging (de Lucia et al., 2017).

By re-analyzing the miRNA microarray data generated by Boon et al. (2013), we also found that miR-23a differentially expressed between older mouse heart and younger mouse heart. miR-23a was firstly identified as a pro-hypertrophic miRNA (Lin et al., 2009). It may convey the hypertrophic signals by suppressing the translation of muscle-specific ring finger protein 1 or regulating the function of Foxo3a (Wang et al., 2012). Lagendijk et al. (2011) found miR-23a was involved in endocardial cell differentiation and migration. More recently, Feldman et al. reported circulating levels of miRNA-23a were significantly decreased in patients with postoperative AF undergoing coronary bypass artery grafting surgery, indicating a significant contribution of miRNA-23a to the development of AF (Feldman et al., 2017). Consistent with this, our study found that expression of miR-23a-5p negatively correlated with human AA, suggesting that miR-23a-5p may play an important role in the pathogenesis of cardiac hypertrophy, AA, and AF.

The remaining five miRNAs are newly identified for human AA in this study. Ishikawa et al. (2017) found that overexpression of miR-1263 accelerated and increased endoderm differentiation. miR-4423 was identified as a primate-specific regulator of airway epithelial cell differentiation and lung carcinogenesis (Perdomo et al., 2013). miR-509-3-5p was identified to have the carcinogenetic role in multiple types of cancers (Vilming Elgaaen et al., 2014; Wang et al., 2016). Few studies have linked miRNA-5683 and miRNA-6514 to cardiovascular diseases or other human diseases. The above results indicated that our analytic pipeline was efficient for detecting miRNAs for human AA. Substantial efforts are required to validate seven AA-miRNAs in studies involving the design of larger sample size and various heart tissues rather than right atria.

Using a similar strategy as we analyzed miRNA-seq data, we identified 23 upregulated and 19 downregulated genes in right atrial tissues of older samples compared to those of younger samples (Figure 3). The expression patterns of 42 AA-genes were highly consistent between two sets of mRNA-seq data (Supplementary Table S5). Previous studies have linked several genes to human aging and cardiovascular diseases. The calcium ion channel *CACNA1G* (L-type calcium voltage-gated channel subunit alpha1G) demonstrated progressively decreased expression with age (Figures 3C,D) (Gan et al., 2013) found that aged left atrial cells showed lower peak L-type calcium currents than normal adult left atrial cells, and both the Cav1.2 mRNA and protein expression levels were reduced in the aged atrial cells. Our results were in line with Gan’s findings. Therefore, the plateau potential was more negative, and the action potential duration was longer in aged atria (Herraiz-Martínez et al., 2015). These age-related changes in L-type calcium channel may facilitate the development of AF. Other genes encoding cardiac cellular structural protein showed differential expression in this study, including *TNNI3* (troponin I 3), *VTN* (vitronectin), *IGHG1* (immunoglobulin heavy constant gamma 1), and *MUC4* (mucin 4). Several genes encoding cellular functional protein were also dysregulated, like *PRKCG* (protein kinase C gamma), *MRPL41* (mitochondrial ribosomal protein L41), and *NAMPT* (nicotinamide phosphoribosyltransferase). The expression level of *IRF1* (interferon regulatory factor 1) showed an age-dependent decrease (Figure 3C) among four age groups, indicating inflammation-related pathological processes might be involved in human AA. In summary, several genes associated with cardiac electrophysiological remodeling, structural remodeling, and inflammatory response demonstrated age-related expression profiles in aged atria, which provides new insight into the transcriptomic architecture of AF.

The major novelty of this study relied on the rigorous integrative analysis of miRNA-seq and mRNA-seq data from the same set of human atrial tissue samples, leading to the discovery of hundreds of miRNA-mRNA interactions underlying human AA. Three AA-miRNAs and eight AA-genes were identified with direct interactions. The most significant miRNA was miR-34a that was well-known to increase age-related cardiomyocyte apoptosis and cardiac dysfunction (Seeger and Boon, 2016). Numerous genes were validated previously as downstream targets of miR-34a, such as sirtuin1, heat shock protein70, vascular endothelial growth factors, vinculin, etc (de Lucia et al., 2017; Seeger and Boon, 2016). We found five direct downstream targets of miR-34a-5p, including *SLITRK3, THBS1, EREG, IRF1*, and *PEG10*. It seemed the miR-34a-5p/IRF1 pair was involved in the regulation of immune responses. Other three pairs (miR-23a-5p/*ADORA3*, miR-509-3-5p/*THBS1*, and miR-509-3-5p/*KLF10*) were also identified in our study, while the possible roles in AA await further studies to reveal. These miRNAs and genes had various indirect interactions with each other, forming complicated regulatory networks for AA (Figure 5). Gene set enrichment analysis showed that rhythmic process is the most important pathway for human AA (Supplementary Table S7). Cardiac rhythm is essential to maintain normal heart function such as heart rate, blood pressure and cardiac muscle contraction (Ivanov et al., 2007). Five genes (*PRKCG, EREG, BHLHE40, KLF10*, and *NAMPT*) targeted by AA-miRNAs may contribute to the pathogenesis of heart arrhythmia such as AF. Rhythmic activation of clock-controlled genes could lead to an oscillation in cardiovascular cells such as fibroblasts, cardiomyocytes (Crnko et al., 2019). TGF-β signaling has been shown to promote cardiac fibrosis during aging (Biernacka and Frangogiannis, 2011). Consistent with this, a recent study showed a pivotal role of TGF-β1 in arrhythmogenesis of the fibrotic heart (Salvarani et al., 2017). Besides, TGF-β2 was found to inhibit leukocyte migration by regulating proinflammatory cytokines (Fabry et al., 1995). In our study, three target genes (*THBS1*, *EREG*, and *IRF1*) of AA-miRNAs were highly enriched in the regulation of cytokine biosynthetic progress. These results suggested that miRNAs might contribute to cardiac aging through multiple physiological pathways. Future studies are needed to clarify the detailed molecular mechanisms between miRNA-mRNA and individual components of these pathways.

miRNA directed interventions were expected to be a promising candidate for the treatment of several cardiovascular diseases. The inhibition of miR-34a through gene deletion or antagomiR was able to reverse both postischemic and age-related cardiac dysfunction (Yang et al., 2015). Anti-miR-34a therapy was found to reduce cardiomyocyte cell death via phosphatase one nuclear targeting subunit and regulation of telomere length. miR-34a had a broad spectrum of downstream targets. Once the abnormal expression or function of miR-34a was rescued, several cardiac cascade reactions might be modified. Therefore, pharmacological modulation of aging-related miRNAs might become a novel strategy to treat AA or AF.

There are several limitations to this study. First, the discovering power of our study is limited due to small sample size. Many patients were excluded in the initial screening using stringent criteria and further selection in order to match baseline demographic and clinical features across four age groups. We anticipate that more AA-related miRNA and genes can be identified in future studies with large sample size. Second, all findings of this study are from human right atrial tissues and may not reflect age-related transcriptomic changes in human left atria (Liu et al., 2014). Third, the identified miRNA-mRNA interactions for AA were mostly based on statistical evidence and predictions from public databases. The causal regulations of each pair and the underlying mechanisms in aging require further functional characterization (Boon et al., 2013).

In conclusion, our study for the first time described miRNA and mRNA expression profiles associated with AA, and identified novel miRNA-mRNA interaction networks and signaling pathways. All these might be helpful to understand pathophysiologic changes of AA or AA-related AF at the transcriptional and post-transcriptional level.

## Data Availability Statement

The datasets GENERATED for this study can be found in the Gene Expression Omnibus https://www.ncbi.nlm.nih.gov/geo/query/acc.cgi?acc=GSE136930.

## Ethics Statement

The studies involving human participants were reviewed and approved by the Ethics Committee of Beijing Anzhen Hospital. The patients/participants provided their written informed consent to participate in this study.

## Author Contributions

YY, CJ, FW, and XT contributed conception and design of the study. YY, FW, HY, and YL organized the database. FW and HY performed the statistical analysis. YY, CJ, and GW wrote the first draft of the manuscript. JW, CZ, JZ, DL, ZZ, and GW wrote the sections of the manuscript. XT and QW revised the sections of the manuscript. All authors contributed to manuscript revision, read, and approved the submitted version.

## Conflict of Interest

The authors declare that the research was conducted in the absence of any commercial or financial relationships that could be construed as a potential conflict of interest.

## Footnotes

1 https://www.r-project.org/

2 https://cytoscape.org/
