# Supplementary material for: Integrative Analysis of miRNA and mRNA Expression Profiles Associated With Human Atrial Aging
Source: Front Physiol. 2019 Sep 19;10:1226. doi: 10.3389/fphys.2019.01226 (PMC6761282; doi:10.3389/fphys.2019.01226)
Supplement: Supplementary file 1 [file Data_Sheet_1.PDF]

Supplemental Figure S1

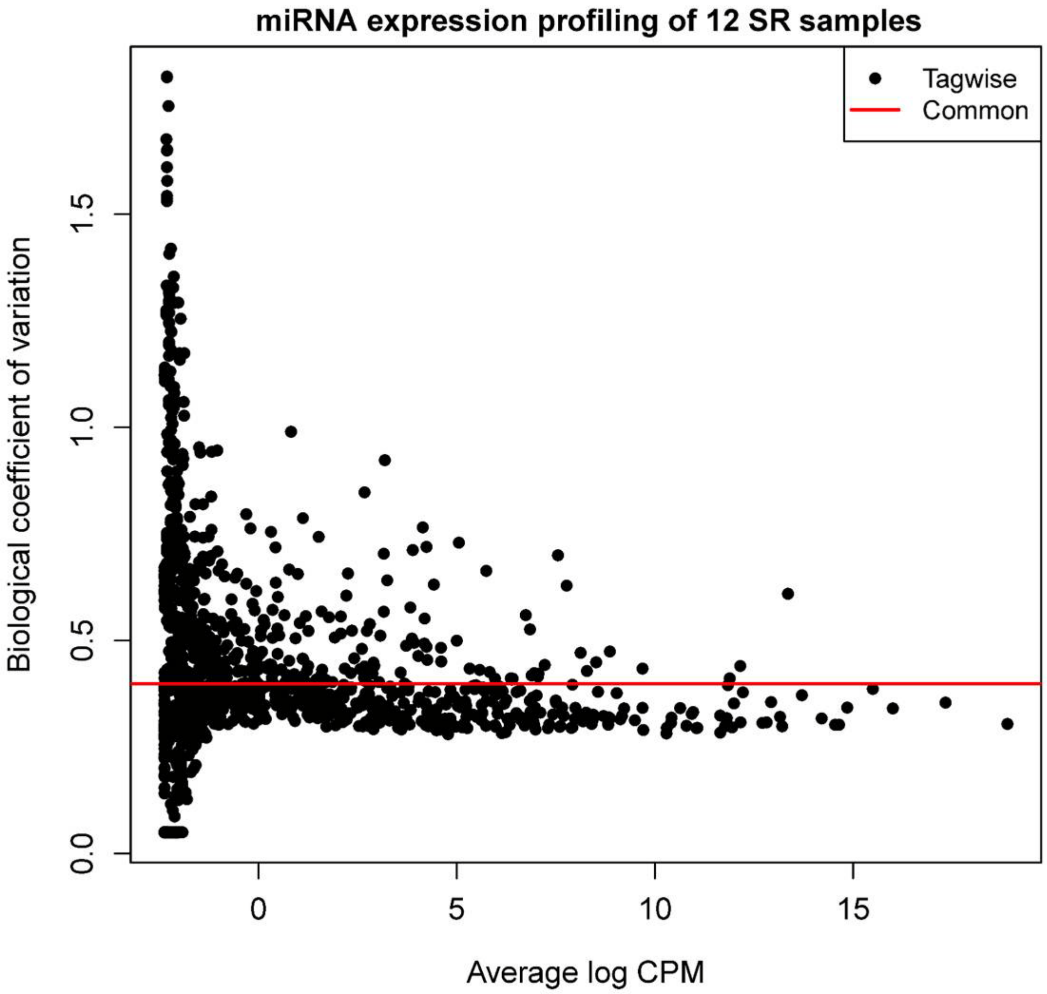

Supplemental Figure S2

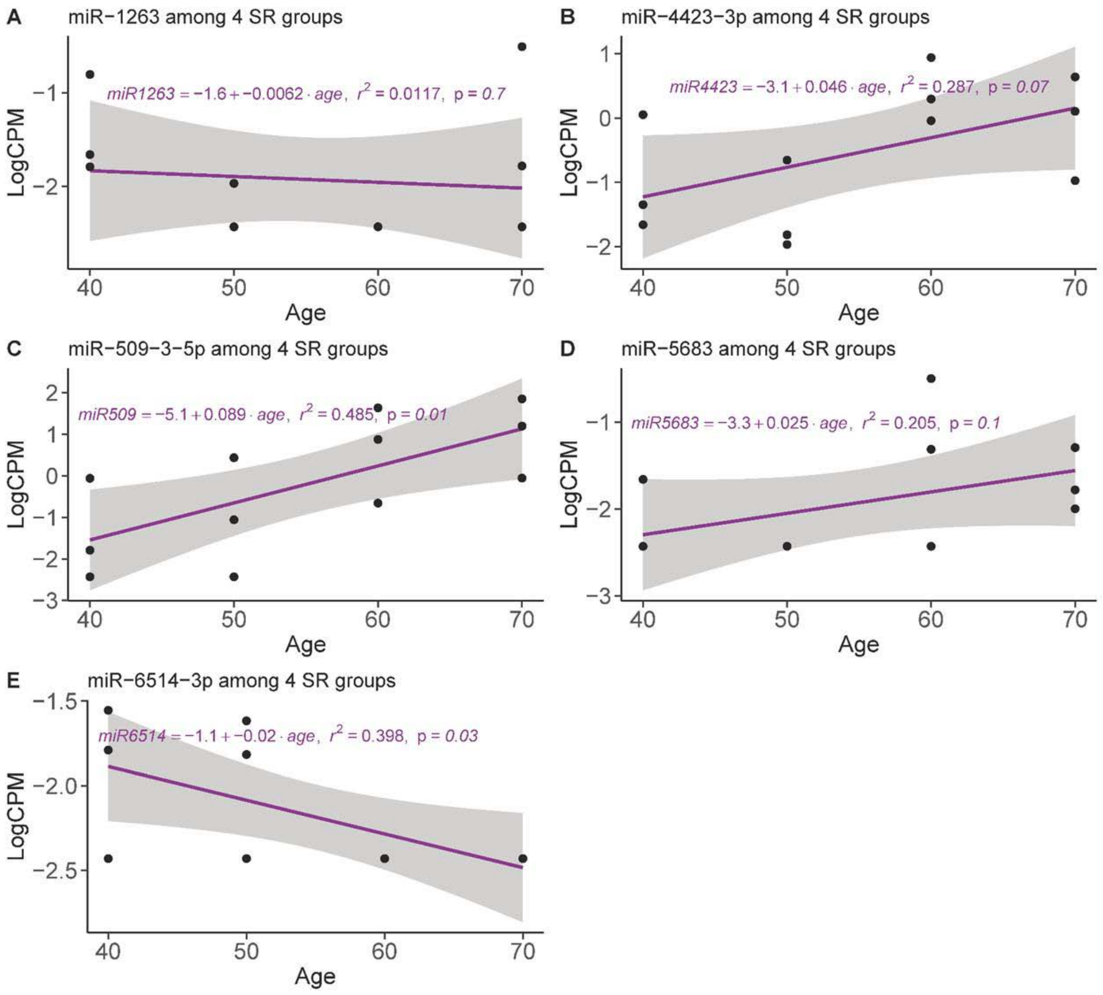

Supplemental Figure S3

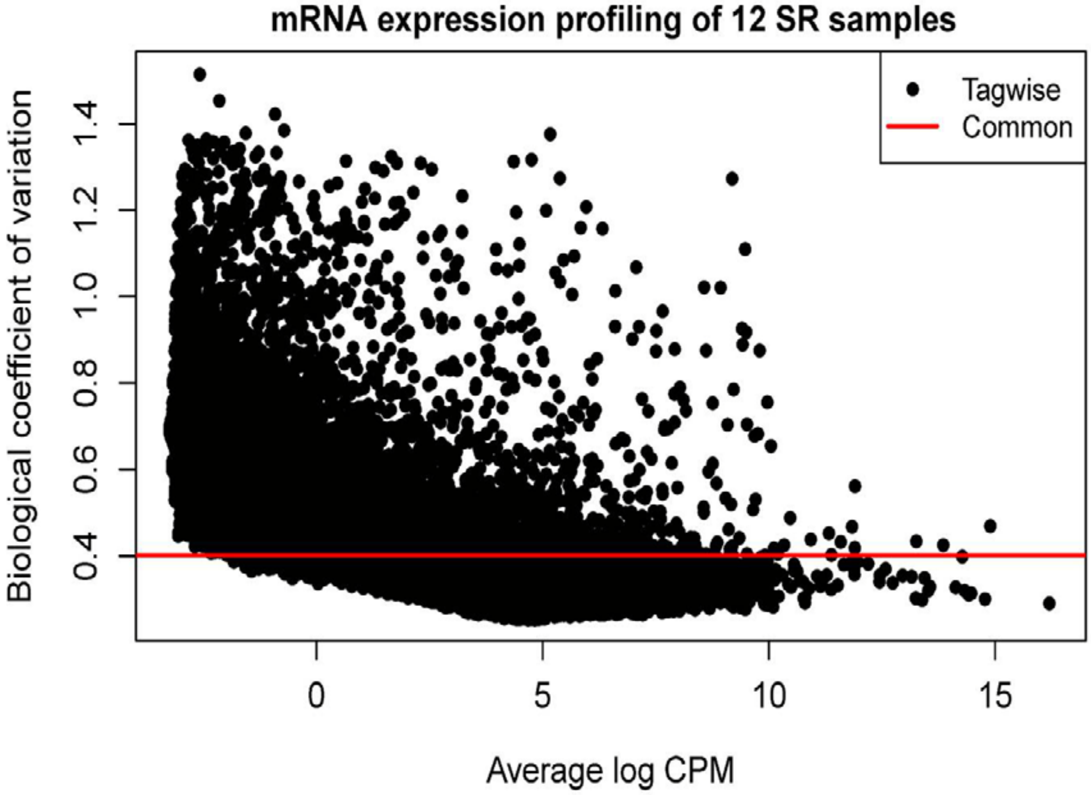

Supplemental Figure S4

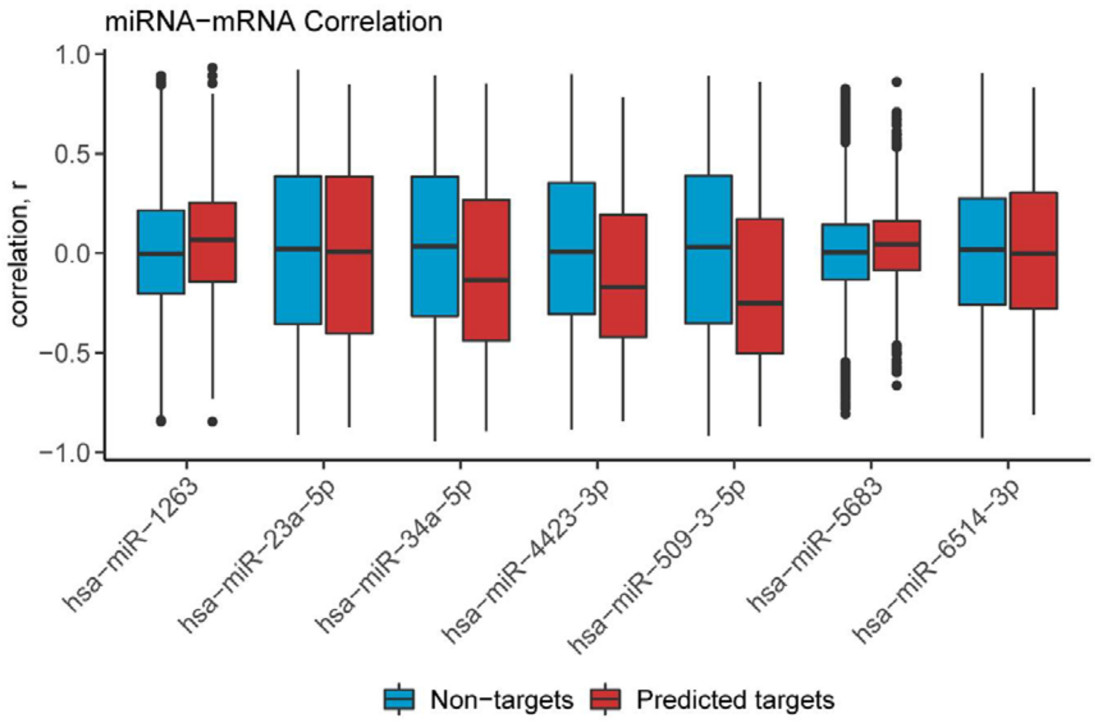

Supplemental Figure Lengds

Figure S1. miRNA expression profiling of 12 SR samples

After normalization of RNA-seq reads, the biological coefficient of variate of each miRNA was plotted against the average expression (denoted as count per million at log2 scale). Red line indicates the Estimate Trended Dispersion for Negative Binomial GLMs. This plot was generated by R package edgeR.

Figure S2. Linear regression analysis of 5 miRNAs with age

The miRNA expression was quantified as count per million at log2 scale (Y-axis). For each miRNA, linear tread and 95% confidence interval were shown as purple line and gray area. **A-E.** Linear regression analysis of 5 miRNAs among four age groups.

Figure S3. mRNA expression profiling of 12 SR samples

After normalization of RNA-seq reads, the biological coefficient of variate of each miRNA was plotted against the average expression (denoted as count per million at log2 scale). Red line indicates the Estimate Trended Dispersion for Negative Binomial GLMs. This plot was generated by R package edgeR.

Figure S4. Pair-wise correlation analysis of 7 AA-miRNAs and 23,346 genes generated from mRNA-seq

For each miRNA, the transcriptome-wide correlation was shown by grouped boxplot. The red color indicated target genes predicted by bioinformatic analysis, implemented by R package multiMiR. Y-axis, Pearson Correlation coefficient.
